# Supplementary material for: Antibacterial Activity of Biodegradable Films Incorporated with Biologically-Synthesized Silver Nanoparticles and the Evaluation of Their Migration to Chicken Meat
Source: Antibiotics (Basel). 2023 Jan 15;12(1):178. doi: 10.3390/antibiotics12010178 (PMC9854460; doi:10.3390/antibiotics12010178)
Supplement: Supplementary file 1 [file antibiotics-12-00178-s001.zip › antibiotics-2146535-supplementary.pdf]

Supplementary Table S1. Antibiotics resistance of *Salmonella* sp. isolates from poultry farms.

**Bacteria**	**Resistance**
S. Saint Paul 08	AMP, PEN, ERY, OXA
S. Saint Paul 09	AMP, PEN, ERY, OXA, AMX, CEF, CET, CRO, CFZ, CTX
S. Saint Paul 10	AMP, PEN, ERY, OXA, NAL, AMX, CEF, CFZ, FOX
S. Saint Paul 11	NAL, PEN, ERY, OXA
S. Saint Paul 12	AMP, PEN, ERY, OXA, AMX, CEF, CET, CRO, CFZ, CTX
S. Saint Paul 13	ERY, OXA
S. Enteritidis 92	NAL, CHL
S. Enteritidis 02	NAL, ERY, OXA
S. Enteritidis 06	AMP, PEN, NAL, ERY, OXA
S. Seftenberg 14	NAL, ERY, OXA
S. Seftenberg 15	NAL, ERY, OXA, AMX, CET, CRO, CFZ, CTX
S. Seftenberg 16	NAL, ERY, OXA
S. Seftenberg 17	NAL, ERY, OXA
S. Seftenberg 18	NAL, ERY, OXA
S. Seftenberg 19	PEN, NAL, ERY, OXA
S. Kentucky 20	AMP, PEN, NAL, ERY, OXA
S. Kentucky 21	PEN, NAL, ERY, OXA
S. Kentucky 22	PEN, NAL, ERY, OXA
S. Kentucky 25	PEN, NAL, ERY, OXA

AMP- ampicillin; AMX – amoxicillin; CEF – cephalothin; CET – ceftiofur; CFZ – cefazolin; CHL-chloramphenicol; CRO- ceftriaxone; CTX – cefotaxime; ERY – erythromycin; FOX – cefoxitin; NAL - nalidixic acid; OXA – oxacillin; PEN – penicillin.
